# Time-dependent transcriptomic response to scrapie exposure in ovine MSC-derived neuron-like cells

**DOI:** 10.3389/fcimb.2026.1938678

**Published:** 2026-09-24

**Authors:** Adelaida Hernaiz, José Gil-Guerrero, Belén Marín, Francisco J. Vázquez, Juan J. Badiola, Pilar Zaragoza, Rosa Bolea, Inmaculada Martín-Burriel

**Affiliations:** 1 Laboratorio de Genética Bioquímica (LAGENBIO), Facultad de Veterinaria, Universidad de Zaragoza, Zaragoza, Spain; 2 Instituto Universitario de Investigación Mixto Agroalimentario de Aragón (IA2) UNIZAR-CITA, Zaragoza, Spain; 3 Instituto de Investigación Sanitaria de Aragón (IIS-Aragón), Zaragoza, Spain; 4 Facultad de Ciencias de la Salud, Universidad San Jorge, Zaragoza, Spain; 5 Centro de Encefalopatías y Enfermedades Transmisibles Emergentes (CEETE), Facultad de Veterinaria, Universidad de Zaragoza, Zaragoza, Spain; 6 Departamento de Patología Animal, Facultad de Veterinaria, Universidad de Zaragoza, Zaragoza, Spain; 7 Centro de Investigación Biomédica en Red de Enfermedades Neurodegenerativas (CIBERNED), Instituto de Salud Carlos III, Madrid, Spain

**Keywords:** early transcriptomic response, neuron-like cells, ovine mesenchymal stem cells, prion, RNA-seq, scrapie

## Abstract

Prion diseases are fatal neurodegenerative disorders in which early molecular responses to prion exposure remain incompletely understood, particularly in naturally susceptible hosts. In the present study, we performed RNA-sequencing analysis of ovine mesenchymal stem cell-derived neuron-like cells exposed to classical scrapie brain homogenate and compared their transcriptomic response with that of cells exposed to normal brain homogenate. Cells were analyzed at 2 and 4 days post-inoculation (dpi). Differential expression analysis identified 76 genes at 2 dpi and 124 genes at 4 dpi, with limited overlap between time points, indicating a dynamic response to scrapie exposure. At 2 dpi, enriched biological processes among upregulated genes were mainly related to signaling- and immune-associated transcriptional responses, whereas downregulated genes were enriched in developmental, morphogenetic and lipid-related processes. At 4 dpi, the transcriptional profile was dominated by broader gene repression and showed reduced functional specificity. RT-qPCR analysis of eight selected genes supported the direction of change detected by RNA-seq in most cases. Overall, these results indicate that scrapie exposure elicits a time-dependent transcriptional response in ovine MSC-derived neuron-like cells and provide an exploratory framework for studying early molecular responses associated with prion exposure in a naturally susceptible species.

## 1 Introduction

Prion diseases, or transmissible spongiform encephalopathies (TSEs), are fatal neurodegenerative disorders affecting humans and animals, including Creutzfeldt-Jakob disease in humans, classical scrapie in sheep and goats, bovine spongiform encephalopathy and chronic wasting disease in cervids. They are associated with the accumulation of misfolded prion protein (PrP^Sc^) and progressive synaptic dysfunction and neuronal loss in the central nervous system (Soto and Satani, 2011; Senatore et al., 2013). Despite extensive research, the precise pathogenic mechanisms involved in prion and prion-like disorders remain incompletely understood and no effective treatments are currently available to halt disease progression (Schlachetzki et al., 2013).

A major challenge in prion research is the limited availability of tractable *in vitro* systems that capture neurotoxic responses while remaining relevant to naturally susceptible hosts and field strains. Classical immortalized cell lines have been helpful to study prion replication and strain properties (Butler et al., 1988; Schätzl et al., 1997; Mahal et al., 2007), however they frequently provide limited information on neurodegenerative endpoints (Krance et al., 2020) and may require host adaptation of prion strains (Butler et al., 1988; Nishida et al., 2000; Klöhn et al., 2003; Mahal et al., 2007). In contrast, primary neuronal cultures and organotypic brain slice cultures can reproduce prion-associated neuropathological features, including neuronal loss and vacuolation (Cronier et al., 2004; Hannaoui et al., 2013; Falsig and Aguzzi, 2008; Falsig et al., 2012; Plug et al., 2024), but their use is restrained by the need for continuous animal sacrifice and limited scalability, particularly in naturally susceptible ruminant species.

From a pathogenesis perspective, prion-associated neurotoxicity can be at least partly dissociated from bulk prion propagation and overt deposition of protease-resistant PrP^Sc^, as early synaptic dysfunction and cellular stress responses often precede, or occur concurrently with initial PrP^Sc^ accumulation, well before robust PrP^Sc^ deposits are detectable by conventional assays (Mallucci, 2009; Mercer and Harris, 2023).

Given that prion-associated neurotoxic signaling requires the presence of PrP^C^, cellular systems that express PrP^C^ but do not support sustained prion replication can still be used to investigate prion-related cellular responses (Resenberger et al., 2011; Chiesa, 2015). Mesenchymal stem cells (MSCs) represent a practical cellular resource from naturally susceptible species, as they can be isolated from accessible tissues, express PrP^C^ (Zhang et al., 2006; Lyahyai et al., 2012), and can be differentiated into neuron-like cells (Pittenger et al., 1999; Zhao et al., 2002). We previously showed that ovine MSCs (oMSCs) do not display detectable infectivity when derived from scrapie-infected sheep (Mediano et al., 2015) and do not propagate PrPSc under standard growth conditions (García‐mendívil et al., 2021), although neuron-like differentiation allows transient maintenance of PrP^Sc^ levels following exposure to scrapie brain material (García‐mendívil et al., 2021; Hernaiz et al., 2023). These observations support the use of differentiated oMSC-derived neuron-like cells as a platform to investigate host- and lineage-dependent molecular responses to prion-associated neurotoxic stimuli.

Here, we apply RNA-seq transcriptomic profiling to ovine oMSC-derived neuron-like cells exposed to classical scrapie brain homogenate and to normal brain homogenate controls, with the aim of characterizing cellular responses to prion-associated stimuli in a naturally susceptible host background.

## 2 Materials and methods

### 2.1 Ovine mesenchymal stem cell culture and neurogenic differentiation

A bone marrow sample was obtained from a two-year-old female Rasa Aragonesa sheep with the ARQ/ARQ genotype for the *PRNP* gene, which is homozygous for Alanine (A), Arginine (R) and Glutamine (Q) at codons 136, 154 and 171, respectively. This genotype is the most commonly found in scrapie-affected sheep of this breed (Acín et al., 2004). Following sedation with xylazine and local anesthesia using lidocaine, a bone marrow aspirate was collected as previously described (García‐mendívil et al., 2021). All procedures were conducted under project license PI44/18, approved by the Ethical Committee for Animal Experiments at the University of Zaragoza. The care and handling of the animals adhered to the Spanish Policy for Animal Protection (RD53/2013), which aligns with European Union Directive 2010/63 on the protection of animals used for experimental and other scientific purposes.

MSCs were isolated from a 2–3 mL bone marrow aspirate using a previously established protocol (Ranera et al., 2012). This method involves separating the mononuclear fraction through density gradient centrifugation with Lymphoprep (STEMCELL Technologies) and subsequently isolating the MSCs based on their ability to adhere to plastic surfaces. After isolation, the cells were expanded to passage 3 in a basal medium composed of low glucose Dulbecco’s Modified Eagle’s Medium (DMEM, Sigma-Aldrich, St. Louis, MO, USA), supplemented with 10% fetal bovine serum (FBS), 1% l-glutamine (Sigma-Aldrich) and 1% streptomycin/penicillin (Sigma-Aldrich).

For the neurogenic differentiation of oMSCs, the HyClone AdvanceSTEM™ Neural Differentiation Kit (ThermoFisher Scientific) was used. The medium was changed every 48 h to maintain the cells in the differentiation state.

The characterization of the oMSC under basal conditions, including their differentiation into mesodermal lineages (adipocytes, chondrocytes and osteocytes), as well as the assessment of their neurogenic differentiation capacity through Nissl bodies staining and neuronal marker analysis, including *MAP2*, *TUBB3* and *NEFM*, was carried out in a previous study (Hernaiz et al., 2023) confirming acquisition of neuron-like characteristics after neural induction. The same MSC donor, differentiation protocol and culture conditions were used in the present study.

### 2.2 Exposure of neuron-like cells to scrapie

Passage 3 cells were seeded at 10,000 cells/cm2 in T25 flasks and maintained under basal conditions for 24 h. Neurogenic differentiation was then induced, reaching its peak after 72 h, as previously described (Mediano et al., 2015). At this stage, neuron-like cells were exposed for 48 h to brain inocula diluted 1:10 in HyClone AdvanceSTEM™ Neural Differentiation medium (ThermoFisher Scientific) and prepared from CNS samples of two sheep: one healthy (normal brain homogenate, DN_NBH) and one naturally infected with classical scrapie (scrapie-treated, DN_SCR), both carrying the ARQ/ARQ genotype for the *PRNP* gene. The inocula were added once at the beginning of the exposure period and remained in the culture medium for the entire 48 h; no additional inocula was added during this period. The inocula were obtained from the tissue bank of the Centre of Encephalopathies and Emerging Transmissible Diseases (CEETE) of the University of Zaragoza.

A total of 18 parallel culture replicates were established from the same passage 3 cell preparation and distributed across the three experimental conditions and two study time points (n = 3 culture replicates per condition and time point). Separate culture replicates were assigned to 2 dpi (days post-inoculation) (T1) and 4 dpi (T2) sampling. After 2 dpi, cells assigned to the first time point were collected, whereas the inocula were removed from the remaining cultures, which were washed twice with PBS and maintained under neurogenic conditions for two additional days until 4 dpi. Neuron-like cells maintained solely in neural differentiation medium (medium only, DN_MO) were used as controls.

### 2.3 RNA extraction of neuron-like cells

For RNA extraction, cells under the different experimental conditions (DN_SCR, DN_NBH and DN_MO) and at the two study time points (2 dpi and 4 dpi) were collected by trypsinization and subsequent centrifugation. Afterwards, RNA was extracted using the Direct-zol RNA MiniPrep kit (Zymo Research) following the manufacturer’s instructions. RNA-seq analysis was performed on three parallel culture replicates per condition and time point.

Before library preparation, RNA integrity was assessed using an Agilent 2100 Bioanalyzer, RNA purity was evaluated by agarose gel electrophoresis and NanoDrop spectrophotometry (ThermoFisher Scientific, Waltham, MA, USA), and RNA quantity was determined using NanoDrop spectrophotometry and the Agilent 2100 Bioanalyzer. Approximately 0.5–1 μg of total RNA per sample was used for RNA-sequencing.

### 2.4 Library preparation and RNA-sequencing

The cDNA libraries were constructed at Novogene in Cambridge (UK). Quantified libraries were pooled and sequenced in a single run on an Illumina platform, generating 150 bp paired-end reads. Quality control of the raw reads was conducted using in-house Perl scripts, and clean reads were obtained after removing reads containing adapters, poly-N sequences, or low-quality bases. The Q20, Q30 and GC content of the clean data were then calculated. No samples were excluded from downstream analyses.

After quality control, paired-end clean reads were aligned to the ovine reference genome oar_rambouillet-v1.0 (Ensembl) using HISAT2 v2.0.5. The mapped reads for each sample were assembled with StringTie v1.3.3b (Pertea et al., 2015) using a reference-based approach. For transcript quantification, FeatureCounts v1.5.0-p3 was used, and FPKM (Fragments Per Kilobase of transcript per Million mapped reads) values were calculated for each gene based on gene length and the number of mapped reads.

### 2.5 Differential gene expression and enrichment analyses

Differential gene expression analysis was conducted using the DESeq2 R package v1.20.0. No additional pre-filtering of low-count genes was applied prior to analysis, and DESeq2 internal normalization and filtering procedures were used. The resulting P-values were adjusted using the Benjamini and Hochberg method to control the false discovery rate (FDR). Genes with an adjusted P-value < 0.05 and a log2(fold change) (log2FC) > 0.58 (upregulated) or < −0.58 (downregulated) were considered differentially expressed (Perez-Roman et al., 2022; Backer et al., 2023; Yan et al., 2023; Hernaiz et al., 2024). Volcano plots of the differentially expressed genes (DEGs) were generated using the SRplot online platform (Tang et al., 2023). Principal component analysis was performed using normalized expression values (log2(FPKM + 1)).

KEGG and Gene Ontology (GO) enrichment analyses of the differentially expressed genes were performed using ShinyGO v0.80 (Ge et al., 2020) to identify biological pathways and biological processes overrepresented among the DEGs relative to the genome background. Analyses were performed separately for upregulated and downregulated gene sets using ovine annotations and the full set of annotated ovine genes as background. KEGG pathways and GO terms with an FDR < 0.05 were considered significantly enriched.

### 2.6 Quantitative real-time PCR

To validate the RNA-sequencing results, a set of eight genes was selected from the significant DEGs identified between scrapie-exposed neuron-like cells (DN_SCR) and cells exposed to normal brain homogenate (DN_NBH) at 2 dpi. Genes were selected based on two criteria: (i) they showed among the largest expression changes in the RNA-seq analysis and (ii) they presented read counts ≥ 1,000. To include both directions of regulation, six upregulated genes and two downregulated genes were chosen for Quantitative real-time PCR (RT-qPCR) validation. This validation step was performed to confirm the expression trends identified by RNA-sequencing. RT-qPCR validation was carried out using the same RNA samples analyzed by RNA-seq, corresponding to three parallel culture replicates per condition.

Reverse transcription was carried out using 200 ng of total RNA with qScript™ cDNA SuperMix (Quanta Biosciences™), following the manufacturer’s instructions. The resulting cDNA was diluted 1:5 in water, and gene expression was quantified by RT-qPCR using Fast SYBR™ Green Master Mix (Applied Biosystems, Thermo Fisher Scientific) on a StepOne Plus Real-Time PCR instrument (Applied Biosystems). Each culture replicate was analyzed in technical triplicate, and the mean Ct value of the three technical replicates was used for downstream calculations. Technical triplicates were used to assess analytical consistency and were not treated as independent observations for statistical analysis.

For RT-qPCR normalization, candidate reference genes were first preselected from the RNA-seq dataset based on relatively high expression and the absence of significant differences between the relevant experimental groups at 2 dpi. Four candidates identified from RNA-seq (*HTATSF1*, *UBAC2*, *VPS41* and *UGGT2*) were then experimentally evaluated together with three commonly used housekeeping genes in ovine scrapie studies (*G6PDH*, *SDHA* and *GAPDH*) (Lyahyai et al., 2010). Gene stability was assessed using the RefFinder web tool (Xie et al., 2023), which integrates geNorm, NormFinder, BestKeeper and the comparative ΔCt method. The three most stable genes, *G6PDH*, *HTATSF1* and *UBAC2*, were used for normalization. Relative expression was calculated using the 2^−ΔΔCt^ method (Livak and Schmittgen, 2001), with the geometric mean of these three reference genes. Mann–Whitney U test was used to identify differences between groups, which were considered significant at P ≤ 0.05. **Table 1** shows the primers used in the RT-qPCR assay.

**Table 1:** Sequences of primers used in the RT-qPCR assay.

Gene	Primer sequences
*HTATSF1*	Fw: CAACCCCCTGAACCCAGT Rv: ACTGTAATGTCTGGAGGCAAA
*UBAC2*	Fw: GTCTTTTCCCTCCTCTGCGT Rv: CCCCTGGAAAACCCCATCTC
*AK4*	Fw: CGTTGGATTCACCCTCCCAG Rv: TCATCCTCCTGCTGGACCAA
*CHAC1*	Fw: TGACAAGATGCCTGGTCGTG Rv: TTGGTATCATAGCCGCCGAG
*CPEB2*	Fw: GGGTCGATTGAGCTATCCACAT Rv: ACCTCGTCTTCGCCCATAAC
*CYP1B1*	Fw: TGGAGACACTGAGATTGCGG Rv: CAGAAAGGTCGGCGACAGAT
*NUFIP2*	Fw: TGACCTGAGGGCTGCTATTG Rv: GGCAGTCTGGTCCTTCATTG
*SMOC1*	Fw: GTCACCGATAAGCCTGCGA Rv: CCGTCATCTGAATTGAGGGT
*UEVLD*	Fw: AGTACCCAGAAAGACCTACTGAAT Rv: GGGGGAGCAAAAGGGTGAG
*ZFP36L2*	Fw: CTTCTGTCCGCCTTCTACGA Rv: CGCCTTCTTGTCCAGCATGT

Fw, Forward primer; Rv, Reverse primer.

## 3 Results

### 3.1 RNA-seq data quality

After filtering, clean reads showed high quality across all samples, with error rates below 1% and more than 95% of bases exceeding Q20. On average, 92% of reads aligned to the ovine reference genome, with 81–89% mapping uniquely and 7–9% mapping to multiple locations. Most reads mapped to exonic regions (67.80% ± 1.37%) (**Supplementary Tables S1**, **S2**). The distribution of gene expression levels was comparable across samples, and high correlation was observed within groups (**Supplementary Figures S1**, **S2**).

### 3.2 Global transcriptomic separation across experimental groups

Principal component analysis revealed a clear segregation between the two experimental time points (**Figure 1**). At 2 days post-inoculation (2 dpi; T1), DN_SCR and DN_NBH samples showed a partial separation along the principal components, whereas at 4 dpi (T2) this separation was reduced, with both groups clustering more closely.

**Figure 1:**
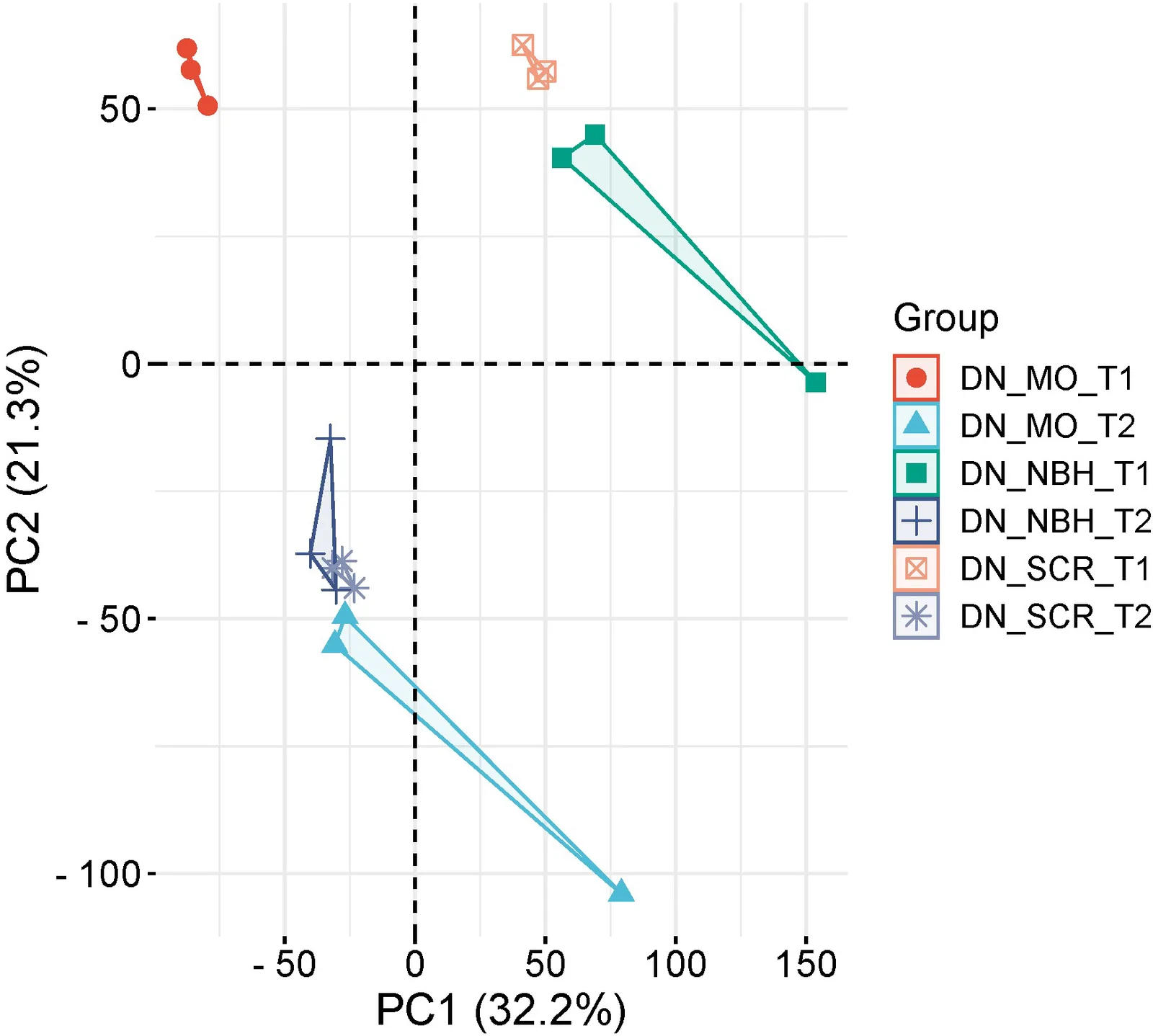
Principal component analysis showing the different time points clustered separately. Cell conditions: DN_MO (Neural Medium Only), DN_NBH (exposed to Normal Brain Homogenate), DN_SCR (exposed to Scrapie brain homogenate). Time point: T1 (2 dpi) and T2 (4 dpi).

### 3.3 Differential gene expression between scrapie and control inoculated cultures

Differential expression analysis between DN_SCR and DN_NBH cells identified 76 differentially expressed genes (DEGs) at T1, including 45 upregulated and 31 downregulated genes (**Table 2**, **Figure 2A**, **Supplementary Table S3**). At T2, 124 DEGs were detected; however, this response was strongly biased toward downregulation, with 14 upregulated and 110 downregulated genes (**Table 2**, **Figure 2B**, **Supplementary Table S4**). All upregulated genes detected at T2 were newly induced at this time point, whereas only seven downregulated genes were shared between both time points (**Figure 2C**).

**Table 2:** Number of differentially expressed genes identified across all compared groups.

Compared groups ^a^	All DEGs ^b^	Up DEGs ^c^	Down DEGs ^d^
DN_SCR_T1 vs DN_NBH_T1	76	45	31
DN_SCR_T2 vs DN_NBH_T2	124	14	110

^a^Compared group: Compared group names.

^b^All DEGs: The total number of differentially expressed genes in the compared groups.

^c^Up DEGs: The upregulated number of differentially expressed genes in the compared groups.

^d^Down DEGs: The downregulated number of differentially expressed genes in the compared groups.

**Figure 2:**
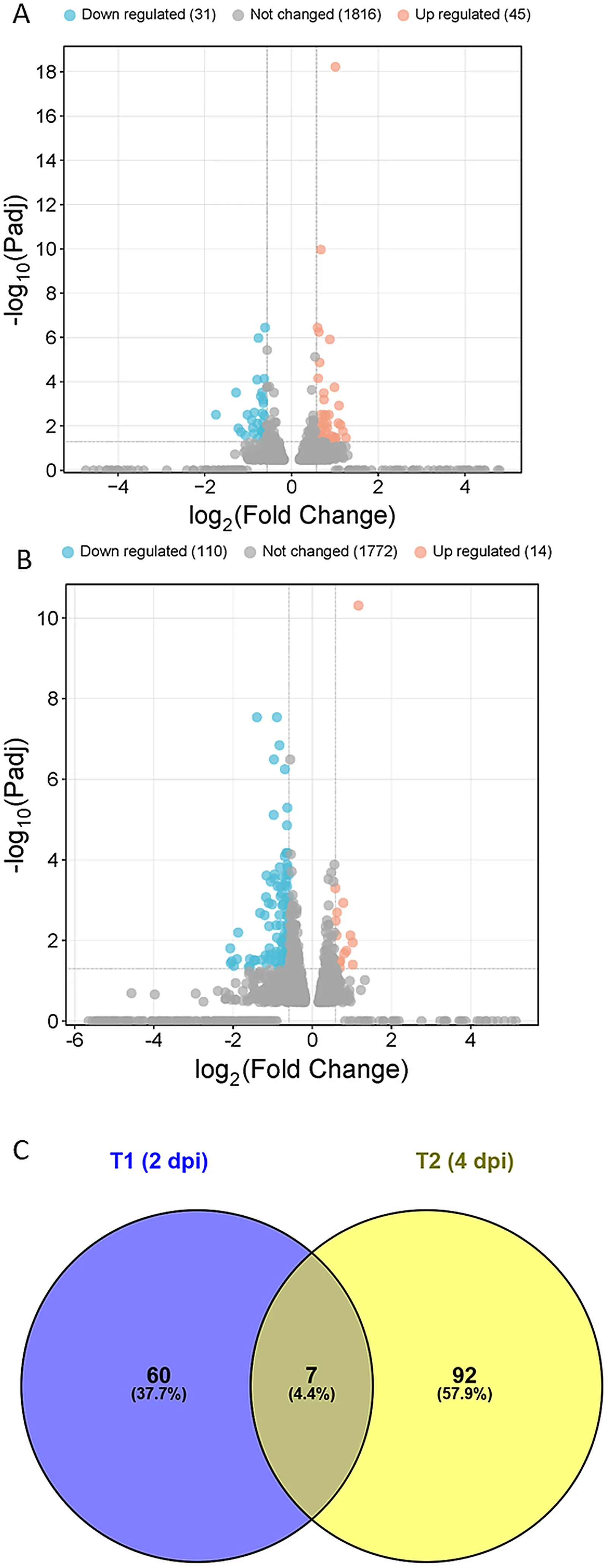
Differential gene expression between DN_SCR and DN_NBH neuron-like cells at T1 and T2. **(A)** Volcano plot showing differentially expressed genes (DEGs) between DN_SCR and DN_NBH cells at 2 days post-inoculation (2 dpi; T1). **(B)** Volcano plot showing DEGs between DN_SCR and DN_NBH cells at 4 dpi (T2). In both plots, the x-axis represents the log2 fold change in gene expression, and the y-axis shows statistical significance (−log10 adjusted p-value). Upregulated genes are shown in orange and downregulated genes in blue. The horizontal dashed line indicates the significance threshold (adjusted p-value < 0.05), and the vertical dashed lines indicate the fold change cut-offs (log2FC > 0.58 and log2FC < −0.58). **(C)** Venn diagram showing the overlap of DEGs identified at T1 (2 dpi) and T2 (4 dpi). The majority of DEGs were specific to each time point, with only seven genes shared between both conditions, all of them downregulated. Percentages indicate the proportion of genes relative to the total number of DEGs identified across both time points.

Volcano plots illustrated these differences, showing a relatively balanced distribution of up- and downregulated genes at T1, in contrast to T2, where the transcriptional response was dominated by gene repression (**Figure 2**).

### 3.4 Functional enrichment analysis of T1 DEGs

Functional enrichment analysis was performed on DEGs identified at T1 (2 dpi) to characterize the biological processes associated with the early transcriptional response to scrapie exposure.

Upregulated genes were significantly enriched in biological processes related to immune system activation and cell signaling, including leukocyte activation and differentiation, immune system development, and regulation of immune responses (**Figure 3A**, **Supplementary Table S5**). In addition, processes linked to cell activation, maintenance of cell number and stem cell population, as well as purine and ribonucleotide metabolic processes, were also enriched among upregulated genes.

**Figure 3:**
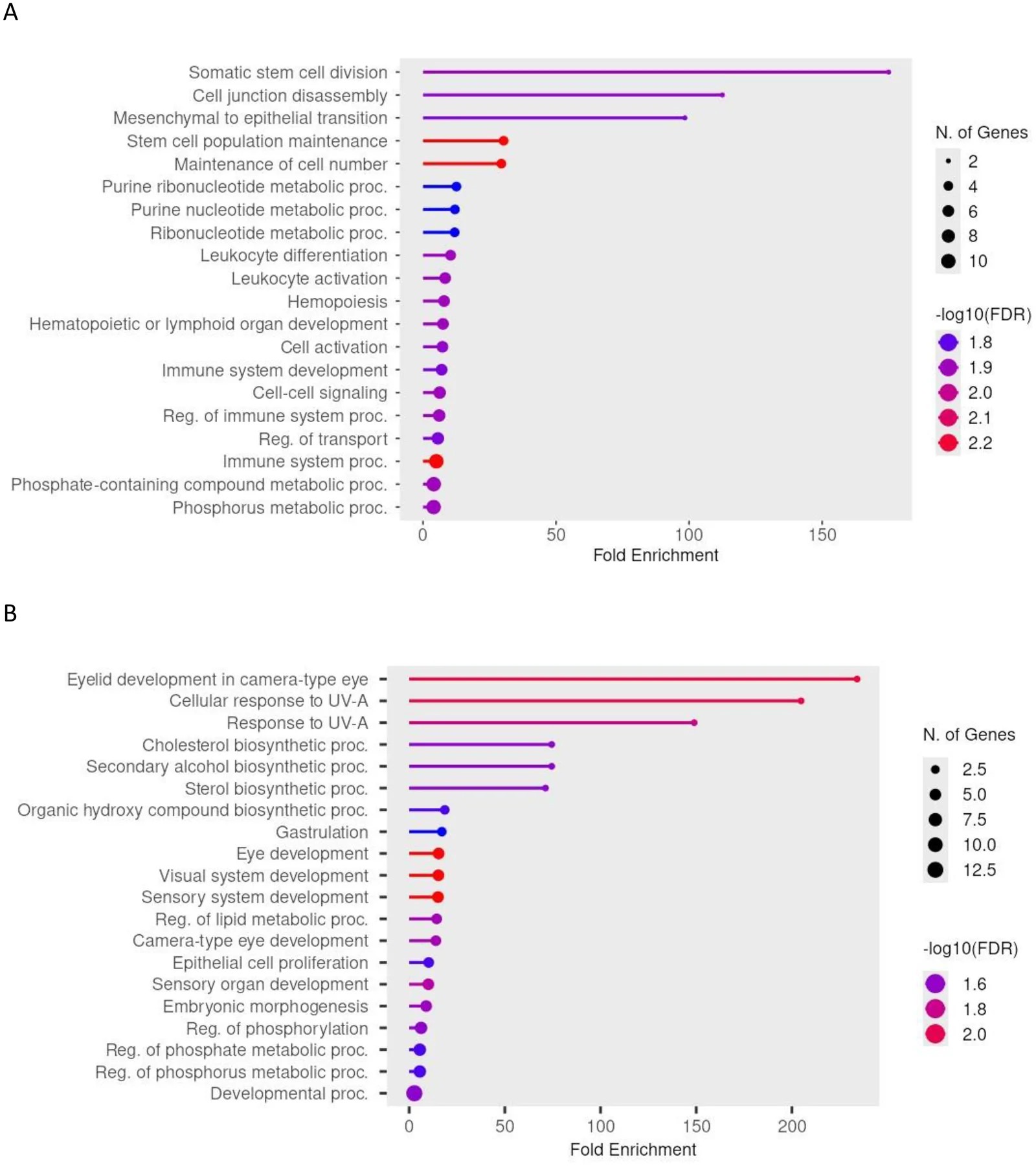
Gene Ontology (GO) biological process enrichment analysis of differentially expressed genes (DEGs) in DN_SCR versus DN_NBH cells at T1 (2 dpi). Enrichment results are shown separately for upregulated **(A)** and downregulated **(B)** genes. The x-axis represents fold enrichment for each GO term, while dot size indicates the number of genes associated with each category and color reflects statistical significance as −log10(FDR).

In contrast, downregulated genes were primarily associated with developmental and morphogenetic processes, including sensory system development, epithelial cell proliferation, and embryonic development (**Figure 3B**, **Supplementary Table S6**). Several metabolic pathways related to lipid, sterol and cholesterol biosynthesis were also enriched among downregulated genes.

These results indicate that early exposure to scrapie is associated with activation of immune- and signaling-related pathways, together with repression of developmental and metabolic programs.

### 3.5 Functional enrichment analysis of T2 DEGs

Functional enrichment analysis of DEGs identified at T2 (4 dpi) revealed a more limited and less specific transcriptional response compared to T1.

Downregulated genes were enriched in broad biological processes related to development and cellular regulation, including anatomical structure development, regulation of signaling, and cellular responses to chemical stimuli (**Figure 4**, **Supplementary Table S7**). However, these categories were relatively general and involved a large number of genes, suggesting a less coordinated functional response at this time point.

**Figure 4:**
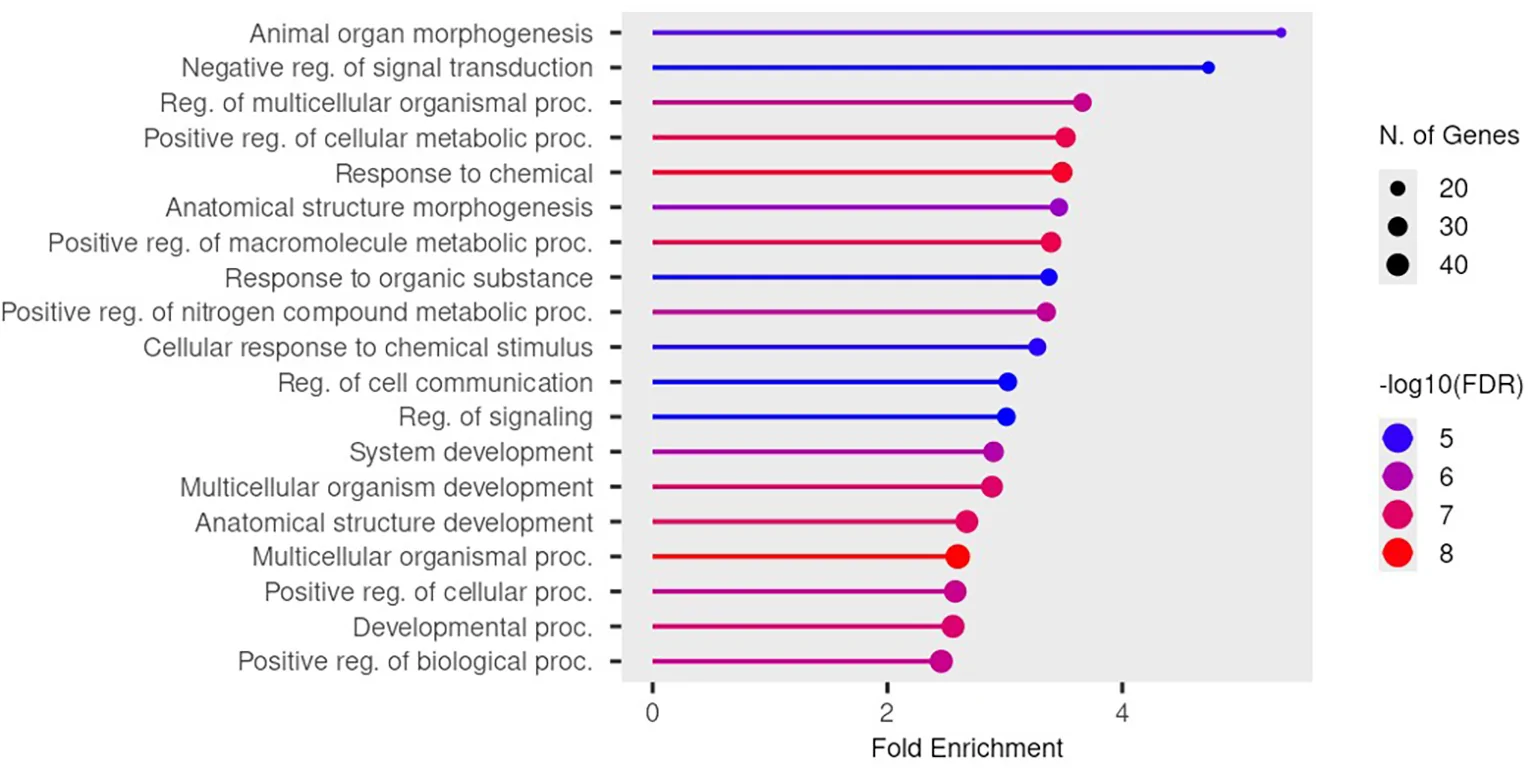
Gene Ontology (GO) biological process enrichment analysis of downregulated differentially expressed genes (DEGs) in DN_SCR versus DN_NBH cells at T2 (4 dpi). The x-axis represents fold enrichment for each GO term, while dot size indicates the number of genes associated with each category and color reflects statistical significance as −log10(FDR).

In contrast, no robust enrichment was detected among upregulated genes at T2, as most categories were supported by very few genes and were therefore not considered biologically meaningful.

These results indicate that the transcriptional response at T2 is characterized by a predominance of gene repression and a reduction in the specificity of enriched biological processes compared to the earlier time point.

### 3.6 Validation of RNA-sequencing results by RT-qPCR

RT-qPCR was performed to assess the expression of eight genes selected from the DEGs identified between DN_SCR and DN_NBH cells at T1. Seven of the eight analyzed genes showed expression changes in the same direction as those detected by RNA-seq (**Table 3**, **Figure 5**). Six genes (*UEVLD*, *ZFP36L2*, *NUFIP2*, *CPEB2*, *AK4* and *CYP1B1*) showed increased expression in DN_SCR cells relative to DN_NBH cells, with differences reaching nominal statistical significance. *CHAC1* showed a concordant downregulation trend, although this difference did not reach statistical significance. In contrast, *SMOC1* did not reproduce the RNA-seq expression pattern. Overall, the RT-qPCR analysis was broadly consistent with the RNA-seq results for most of the selected genes.

**Table 3:** RT-qPCR analysis of selected genes identified as differentially expressed in the DN_SCR versus DN_NBH comparison at T1.

Gene	2^-ΔΔCt^ DN_SCR_T1	2^-ΔΔCt^ DN_NBH_T1	RT-qPCR direction	RNAseq direction	Concordant trend	P-value
*UEVLD*	1.97	1	Up	Up	Yes	0.05
*ZFP36L2*	2.33	1	Up	Up	Yes	0.05
*NUFIP2*	1.87	1	Up	Up	Yes	0.05
*CPEB2*	1.83	1	Up	Up	Yes	0.05
*AK4*	1.90	1	Up	Up	Yes	0.05
*SMOC1*	1.15	1	Up	Down	No	0.35
*CYP1B1*	1.85	1	Up	Up	Yes	0.05
*CHAC1*	0.73	1	Down	Down	Yes	0.10

Relative expression changes were calculated using the 2^-ΔΔCt^ method. Concordance indicates whether the direction of change observed by RT-qPCR matched that detected by RNA-seq.

**Figure 5:**
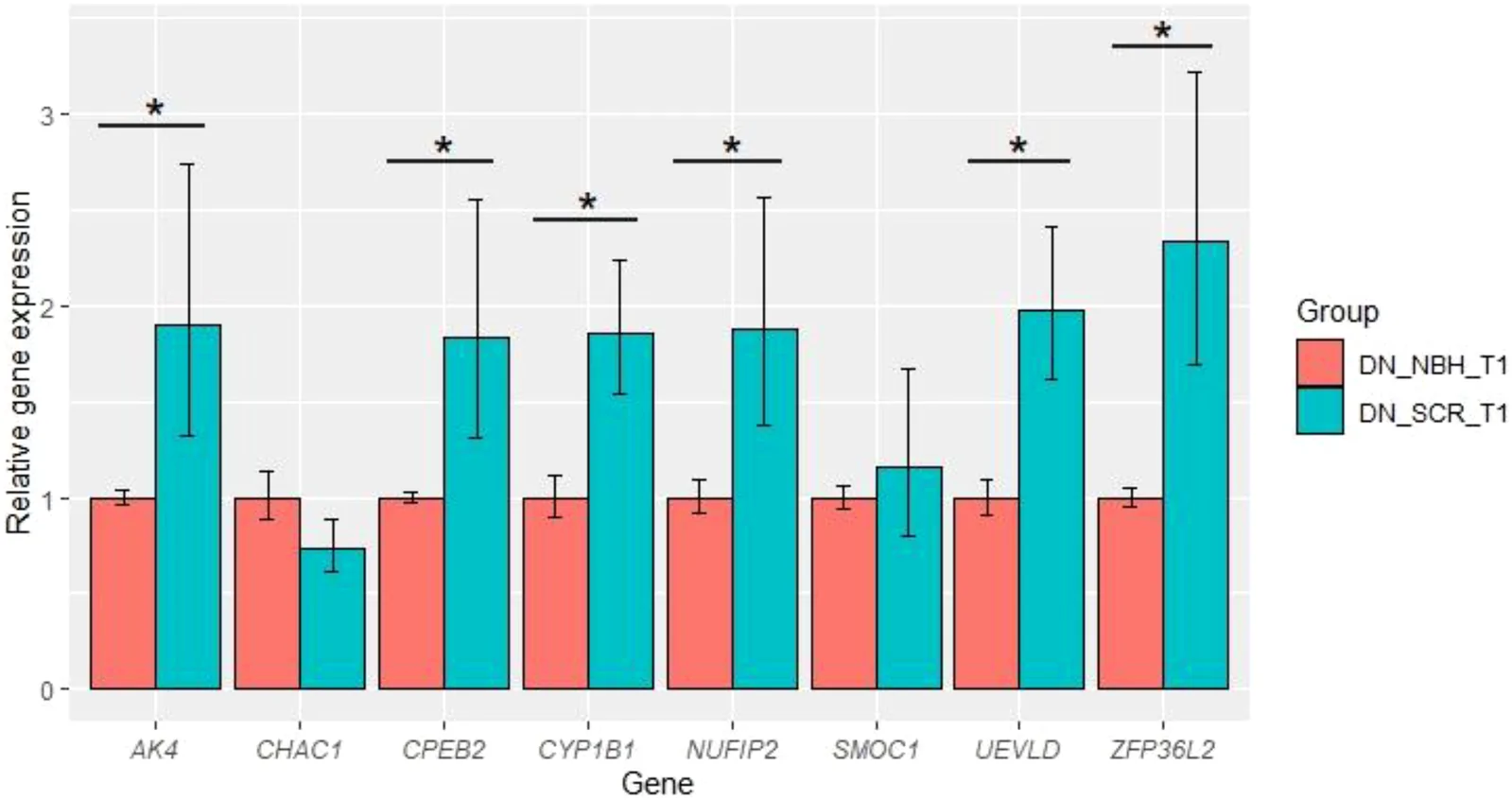
RT-qPCR analysis of selected DEGs identified at T1 in the DN_SCR versus DN_NBH comparison. Relative expression levels are shown as 2^-ΔΔCt^ mean values ± standard error (SE) for scrapie-exposed neuron-like cells (DN_SCR_T1, blue bars) and neuron-like cells exposed to normal brain homogenate (DN_NBH_T1, red bars) at 2 dpi. *P ≤ 0.05.

## 4 Discussion

In the present study, RNA-seq analysis of oMSC-derived neuron-like cells exposed to scrapie brain homogenate revealed a time-dependent transcriptional response when compared with cells exposed to normal brain homogenate. The most evident enrichment signal was observed at 2 dpi (T1), whereas by 4 dpi (T2) the transcriptional profile was dominated by broader gene repression and showed reduced functional specificity. These findings indicate that the molecular responses associated with scrapie exposure in differentiated ovine neuron-like cells vary over time.

At T1, enriched processes related to immune signaling, leukocyte activation, and regulation of cellular responses indicate the activation of early immune- and stimulus-associated transcriptional programs in scrapie-exposed neuron-like cells. These changes are consistent with modulation of complement-, cytokine-, and stress-related signaling networks, aligning with previous reports of early prion-associated immune responses (Li et al., 2021; Wright et al., 2024; Crespo et al., 2012; Aguzzi and Zhu, 2017). At a broader level, the observed transcriptional profile supports the engagement of coordinated signaling modules involved in innate immune regulation, inflammatory signaling, and cellular adaptation processes. Such responses have been previously linked to early stages of prion pathogenesis, including mechanisms associated with glial regulation, tissue repair, and cell survival (Michel et al., 2013; Hartmann et al., 2019; Cianciulli et al., 2020; Davis and Pennypacker, 2017). Overall, these findings are consistent with an early adaptive response in oMSC-derived neuron-like cells, reflecting conserved immune-regulatory and stress-responsive pathways described in prion-related contexts.

In addition to these signaling-related changes, several differentially expressed genes at T1 were linked to post-transcriptional regulation and rapid cellular adaptation. In particular, *ZFP36L2* and *CPEB2* are involved in the control of mRNA stability and translation (Adachi et al., 2014; Lu et al., 2024). Their regulation is compatible with an early response involving post-transcriptional adjustment and signaling fine-tuning. This interpretation agrees with evidence that prion diseases alter RNA metabolism and gene expression regulation at early stages (Lenzken et al., 2014; Sorce et al., 2020). In the context of the present study, such early regulatory changes may be relevant to the broader predominance of gene downregulation observed at T2.

Concurrently, downregulated genes at T1 were enriched in developmental and morphogenetic processes, including sensory system development, anatomical structure formation, and epithelial cell proliferation. These enrichments suggest coordinated alterations in gene networks governing cellular identity, structural organization, and differentiation state. In this context, the observed transcriptional changes are consistent with reduced signaling through pathways related to Wnt activity, cell adhesion, and the activin/TGF-β superfamily. In the nervous system, these pathways are known to regulate neuronal differentiation, axon guidance, synapse formation, synaptic plasticity, and neuroprotective responses (Pascual-Vargas and Salinas, 2021; László and Lele, 2022; Link et al., 2016). Taken together, these findings support an early perturbation of programs associated with structural maintenance and differentiated cellular function.

Another notable aspect of the early response was the enrichment of lipid-, sterol- and cholesterol-related processes among downregulated genes. The reduced expression of genes points to changes in membrane-associated metabolic pathways. This is relevant in the context of prion diseases, as cholesterol-rich membrane microdomains influence prion protein localization, trafficking and conversion, and disturbances in lipid homeostasis are increasingly recognized as contributors to protein misfolding disorders (Lewis and Hooper, 2011; Vendruscolo, 2022). Although this model is not designed to reproduce sustained prion replication, the observed transcriptional changes indicate that scrapie-associated stimuli can affect pathways relevant to membrane homeostasis and differentiated cell function.

At the later time point (T2), the transcriptional response differed from that observed at T1. Differential expression was strongly biased toward downregulation, and the overlap between the DEG sets at both time points was limited. In agreement with this, functional enrichment analysis of T2 downregulated genes identified broader and less specific biological processes, such as regulation of signaling and cellular responses to chemical stimuli. By contrast, no robust enrichment was detected among upregulated genes at T2, as most categories were supported by very few genes and were therefore not considered biologically meaningful. Taken together, these findings indicate that the later stage is characterized by a transcriptional profile dominated by gene repression and reduced functional specificity. The limited overlap between T1 and T2 further suggests that the later response is not simply an extension of the earlier one.

Nevertheless, the predominance of downregulated genes observed at 4 dpi should be interpreted with caution, as cell viability and cytotoxicity were not directly assessed in the present experiment. Although previous studies using the same ovine MSC-derived neuron-like cell model demonstrated maintenance of cell viability following scrapie exposure (Hernaiz et al., 2023), the possibility that cellular stress or toxicity contributed to part of the transcriptional response observed at the later time point cannot be completely excluded. Future studies combining transcriptomic analyses with concurrent viability and cytotoxicity measurements would help clarify this aspect.

Several genes were persistently downregulated at both time points, including *MMP9*, *PENK*, *COL8A2*, *TH*, *BGLAP*, *PARM1* and *HID1*. Among them, *TH* is of particular interest, as its reduced expression is compatible with altered maintenance of differentiation-associated and neuron-like features. This interpretation is consistent with the concurrent reduction of signaling modules linked to Wnt, activin/TGF-β and cell adhesion, which are involved in neuronal differentiation, structural organization and specialized function (Link et al., 2016; Pascual-Vargas and Salinas, 2021; László and Lele, 2022). In parallel, the downregulation of *MMP9* may indicate reduced extracellular matrix remodeling activity. Given the roles of MMP9 in synaptic plasticity, extracellular matrix turnover and inflammatory tissue remodeling in the nervous system (Stawarski et al., 2014), this change is compatible with a less dynamic extracellular environment in scrapie-exposed cultures.

These observations differ from those obtained in our previous transcriptomic analysis of undifferentiated ovine MSCs exposed to the same inoculum (Hernaiz et al., 2024), where the response was dominated by pathways related to proliferation, extracellular matrix remodeling and inflammatory modulation rather than by changes in developmental programs. Although the two models are not directly comparable at a statistical level because of differences in cellular state and experimental context, together they suggest that neurogenic differentiation modifies the transcriptional response to scrapie-associated stimuli. In this regard, the present results indicate that the differentiated state is associated with responses affecting signaling, differentiation-related programs and lipid homeostasis that were not prominent in undifferentiated MSCs.

RT-qPCR analysis supported the RNA-seq results for most of the selected genes, with concordant expression trends observed in seven out of eight cases. Six genes showed statistically significant changes, whereas *CHAC1* displayed a concordant but non-significant downregulation trend. These results should be interpreted mainly as technical support for the direction of change detected by RNA-seq.

From a broader perspective, this model provides a tractable platform for exploring early molecular responses associated with prion exposure in a naturally susceptible species. Unlike systems focused on productive prion replication, oMSC-derived neuron-like cells allow the investigation of prion-associated cellular responses in a differentiated context where PrP^C^ is expressed and PrP^Sc^ remains detectable after exposure, whereas sustained propagation has not been established under standard conditions. Previous work from our group using the same cell model and experimental conditions showed that PrP^Sc^ signal declines over time in oMSC cultures maintained under growth conditions, while neuron-like differentiated cultures retain detectable PrP^Sc^ following inoculation (García‐mendívil et al., 2021; Hernaiz et al., 2023). Although PrP^Sc^ detection was not directly performed in the samples analyzed in the present transcriptomic study and therefore its presence is inferred from previous characterization of the model rather than experimentally confirmed here, this makes the model suitable for studying early signaling alterations, changes in differentiation-associated programs and metabolic responses associated with scrapie exposure. In addition, the possibility of comparing undifferentiated MSCs and neuron-like differentiated cells within the same species provides a useful framework to explore lineage-dependent responses to scrapie-associated stimuli.

Several limitations should be acknowledged. Brain homogenates are complex biological materials that contain, in addition to misfolded prion protein, other molecules capable of influencing cellular responses. Therefore, the transcriptional changes observed here should be interpreted as scrapie-associated rather than exclusively attributable to PrP^Sc^ itself. In addition, the experimental design relied on oMSC cultures derived from a single donor animal and on scrapie and control brain homogenates obtained from one animal each. As a result, the DN_SCR versus DN_NBH comparison may partly reflect donor-specific properties of the inocula in addition to infection status, and the findings should therefore be considered exploratory within this experimental system. This limitation is particularly relevant when interpreting individual DEGs and enriched pathways, as some of the observed transcriptional changes could be influenced by biological characteristics specific to the donor animals used for cell isolation and inoculum preparation. Consequently, the molecular signatures identified in this study should be regarded as exploratory and hypothesis-generating rather than definitive markers of scrapie exposure. Future studies including multiple cell donors and independent inocula will be required to determine the reproducibility and generalizability of these transcriptomic responses. Likewise, this model is not intended to reproduce sustained prion replication, but rather to capture early molecular responses to exposure in a differentiated ovine cellular context.

It is also important to acknowledge that RT-qPCR validation was restricted to genes identified at T1, which was selected because it showed the clearest functional enrichment patterns and the most biologically interpretable response. Nevertheless, the absence of experimental validation of T2-specific DEGs represents a limitation of the present study and future investigations should assess the reproducibility of the transcriptional changes detected at the later time point.

Furthermore, the use of three biological replicates per group limits statistical power and may have reduced sensitivity to detect modest transcriptional changes. Consequently, some biologically relevant effects may have remained undetected, and independent validation in larger datasets will be necessary.

To conclude, differentiated ovine oMSC-derived neuron-like cells exposed to scrapie display a time-dependent transcriptional response characterized by early changes in signaling- and immune-associated pathways together with repression of developmental and metabolic programs, followed by a later stage dominated by broader gene downregulation and reduced functional specificity. Within the limitations of this experimental system, these findings provide an exploratory transcriptomic characterization of molecular responses associated with scrapie exposure and highlight the importance of cellular context in shaping the transcriptional response to prion-associated stimuli. Further studies using additional biological donors and inocula will be necessary to determine the extent to which these changes are reproducible and representative of scrapie-associated responses beyond this model.

## Data Availability

The data presented in this study are available in this published article, its supplementary information files and in NCBI’s Gene Expression Omnibus (Edgar et al., 2002) (GEO) repository through GEO Series accession number GSE287683 (https://www.ncbi.nlm.nih.gov/geo/query/acc.cgi?Acc=GSE287683).
