# Supplementary material for: Time-dependent transcriptomic response to scrapie exposure in ovine MSC-derived neuron-like cells
Source: Front Cell Infect Microbiol. 2026 Sep 24;16:1938678. doi: 10.3389/fcimb.2026.1938678 (PMC13645588; doi:10.3389/fcimb.2026.1938678)
Supplement: Supplementary file 1 [file Table1.docx]

Supplementary Material

# 1 Supplementary Figures and Tables

## 1.1 Supplementary Figures

**Supplementary Figure S1.** Sample gene expression distribution box plot. X axis represents the name of the sample. Y axis indicates the log2(FPKM+1). Parameters of box plots are indicated, including maximum, upper quartile, mid-value, lower quartile and minimum.

**Supplementary Figure S2.** Inter-sample correlation heat map. R^2^ = Square of Pearson correlation coefficient (R).

## 1.2 Supplementary Tables

**Supplementary Table S1.** Data quality control summary.

| **Sample^a^** | **raw_reads^b^** | **raw_bases^c^** | **clean_reads^d^** | **clean_bases^e^** | **error_rate^f^** | **Q20^g^** | **Q30^h^** | **GC_pct^i^** |
| --- | --- | --- | --- | --- | --- | --- | --- | --- |
| DN_MO_T1_1 | 84190260 | 12.63G | 83213834 | 12.48G | 0.04 | 92.82 | 84.84 | 51.93 |
| DN_MO_T1_2 | 88685198 | 13.3G | 87734114 | 13.16G | 0.04 | 92.71 | 84.63 | 51.91 |
| DN_MO_T1_3 | 84575246 | 12.69G | 83580254 | 12.54G | 0.04 | 92.48 | 84.29 | 52.15 |
| DN_SCR_T1_1 | 81949638 | 12.29G | 80910350 | 12.14G | 0.04 | 92.82 | 84.87 | 51.87 |
| DN_SCR_T1_2 | 83711148 | 12.56G | 82659498 | 12.4G | 0.04 | 92.69 | 84.64 | 52.28 |
| DN_SCR_T1_3 | 103505480 | 15.53G | 102168230 | 15.33G | 0.04 | 92.61 | 84.57 | 51.85 |
| DN_NBH_T1_1 | 84507528 | 12.68G | 83438276 | 12.52G | 0.04 | 92.61 | 84.57 | 52.24 |
| DN_NBH_T1_2 | 83825368 | 12.57G | 82841170 | 12.43G | 0.04 | 92.27 | 83.96 | 52.05 |
| DN_NBH_T1_3 | 108115402 | 16.22G | 106834454 | 16.03G | 0.04 | 92.7 | 84.64 | 51.61 |
| DN_MO_T2_1 | 85055228 | 12.76G | 84049780 | 12.61G | 0.03 | 96.92 | 91.89 | 51.28 |
| DN_MO_T2_2 | 90879816 | 13.63G | 90013456 | 13.5G | 0.03 | 97.48 | 92.98 | 52.45 |
| DN_MO_T2_3 | 115433284 | 17.31G | 114378394 | 17.16G | 0.03 | 97.68 | 93.46 | 52.15 |
| DN_SCR_T2_1 | 95907830 | 14.39G | 95107110 | 14.27G | 0.03 | 97.58 | 93.24 | 52.75 |
| DN_SCR_T2_2 | 85198882 | 12.78G | 84504510 | 12.68G | 0.03 | 97.69 | 93.48 | 52.56 |
| DN_SCR_T2_3 | 103439846 | 15.52G | 102596846 | 15.39G | 0.03 | 97.64 | 93.41 | 52.97 |
| DN_NBH_T2_1 | 84135642 | 12.62G | 83404688 | 12.51G | 0.03 | 97.49 | 93.06 | 52.63 |
| DN_NBH_T2_2 | 86435666 | 12.97G | 85684466 | 12.85G | 0.03 | 97.43 | 92.93 | 53.62 |
| DN_NBH_T2_3 | 86184252 | 12.93G | 85282616 | 12.79G | 0.03 | 97.61 | 93.37 | 53.88 |

^a^ Sample: Sample name.

^b^ Raw reads: Reads count from the raw data, four rows as a unit, with statistics of reads count for every sequencing.

^c^ Raw bases: Base number of raw data. (number of raw reads) * (sequence length), converting unit to G.

^d^ Clean reads: Base number of raw data after filtering. (number of clean reads) * (sequence length), converting unit to G.

^e^ Clean bases: (clean base=clean reads*150bp) number multiply read length, saved in G unit.

^f^ Error rate: Average sequencing error rate, which is calculated by Qphred=-10log10(e).

^g^ Q20: The percentage of the bases whose Q Phred values is greater than 20. (Number of bases with Q Phred value > 20) / (Number of total bases) *100.

^h^ Q30: The percentage of the bases whose Q Phred values is greater than 30. (Number of bases with Q Phred value > 30) / (Number of total bases) *100.

^i^ GC pct: The percentage of G&C base numbers of total bases. (G&C base number) / (Total base number)*100.

**Supplementary Table S2.** Comparison of sample and reference genomes.

| **Sample^a^** | **total_reads^b^** | **total_map^c^** | **unique_map^d^** | **multi_map^e^** |
| --- | --- | --- | --- | --- |
| DN_MO_T1_1 | 83213834 | 73581193(88.42%) | 67862168(81.55%) | 5719025(6.87%) |
| DN_MO_T1_2 | 87734114 | 77387304(88.21%) | 71477671(81.47%) | 5909633(6.74%) |
| DN_MO_T1_3 | 83580254 | 73310261(87.71%) | 67797355(81.12%) | 5512906(6.6%) |
| DN_SCR_T1_1 | 80910350 | 71606960(88.5%) | 65994523(81.56%) | 5612437(6.94%) |
| DN_SCR_T1_2 | 82659498 | 72954039(88.26%) | 66994439(81.05%) | 5959600(7.21%) |
| DN_SCR_T1_3 | 102168230 | 89865688(87.96%) | 82497924(80.75%) | 7367764(7.21%) |
| DN_NBH_T1_1 | 83438276 | 73465851(88.05%) | 67653680(81.08%) | 5812171(6.97%) |
| DN_NBH_T1_2 | 82841170 | 72371882(87.36%) | 66808470(80.65%) | 5563412(6.72%) |
| DN_NBH_T1_3 | 106834454 | 94425614(88.38%) | 86985678(81.42%) | 7439936(6.96%) |
| DN_MO_T2_1 | 84049780 | 80749046(96.07%) | 73662839(87.64%) | 7086207(8.43%) |
| DN_MO_T2_2 | 90013456 | 86906794(96.55%) | 79028810(87.8%) | 7877984(8.75%) |
| DN_MO_T2_3 | 114378394 | 110665660(96.75%) | 100687872(88.03%) | 9977788(8.72%) |
| DN_SCR_T2_1 | 95107110 | 91890204(96.62%) | 83879321(88.19%) | 8010883(8.42%) |
| DN_SCR_T2_2 | 84504510 | 81761938(96.75%) | 73936980(87.49%) | 7824958(9.26%) |
| DN_SCR_T2_3 | 102596846 | 99168706(96.66%) | 91385305(89.07%) | 7783401(7.59%) |
| DN_NBH_T2_1 | 83404688 | 80661215(96.71%) | 73457170(88.07%) | 7204045(8.64%) |
| DN_NBH_T2_2 | 85684466 | 82723003(96.54%) | 75841463(88.51%) | 6881540(8.03%) |
| DN_NBH_T2_3 | 85282616 | 82388864(96.61%) | 74575464(87.45%) | 7813400(9.16%) |

^a^ Sample: Sample name.

^b^ Total reads: Total clean reads used for analysis.

^c^ Total map: Number and percentage of reads aligned to the genome, total mapping rate: (mapped reads)/(total reads)*100.

^d^ Unique map: Number and percentage of reads aligned to the unique position of the reference genome, unique mapping rate: (uniquely mapped reads)/(total reads)*100.

^e^ Multi map: Number and percentage of reads aligned to multiple locations in the reference genome, multiple mapping rate: (multiple mapped reads)/(total reads)*100.
